# Pre-Myopic Children: Trends in Myopia Development and Management in Canada

**DOI:** 10.3390/jcm15072748

**Published:** 2026-04-05

**Authors:** Amy H. Y. Chow, Barbara Caffery, Angela Di Marco, Sarah Guthrie, Mira Acs, Stephanie Fromstein, Shalu Pal, Stephanie Ramdass, Vishakha Thakrar, Matthew Zeidenberg, Deborah A. Jones

**Affiliations:** 1 School of Optometry and Vision Science, University of Waterloo, 200 Columbia Street West, Waterloo, ON N2L 3G1, Canada; sarah.guthrie@uwaterloo.ca (S.G.);; 2 Centre for Eye and Vision Research (CEVR), 17W Hong Kong Science Park, Hong Kong SAR, China; 3 Toronto Eye Care, Toronto, ON M4W 1A5, Canadaangela.dimarco.od@gmail.com (A.D.M.);; 4 Dr. Shalu Pal & Associates—Yorkville Eye Institute, Toronto, ON M5S 2V1, Canada; 5 Eyeacademy.ca, 800 Southdown Rd Unit A2, Mississauga, ON L5J 2Y4, Canada; 6 Vaughan Vision Centre, Toronto, ON L4K 0H2, Canada

**Keywords:** pre-myopia, hyperopic reserve, myopia development, retrospective review

## Abstract

**Background/Objectives**: Given the growing prevalence of myopia worldwide, prevention and proactive management of at-risk children becomes increasingly important. This study sought to evaluate trends in myopia development in pediatric pre-myopic patients and determine how optometrists in Canada manage pre-myopia. **Methods**: In this retrospective chart review, records for children aged 6–10 years who had an eye exam between 2017 and 2021 were reviewed. Pre-myopic children were included if the presenting refraction at the first visit was between +0.75D and −0.25D (inclusive). Up to five unique patients were selected for each age (6, 7, 8, 9, and 10) and initial visit year (2017 to 2021) at each clinical site. Demographic information, refractive status and recommended interventions were recorded. **Results**: A total of 1740 pre-myopic patients were included across 15 practices in Ontario, of which 184 patients developed myopia (10.6%) during the years studied. Cohort year groups did not differ in baseline age (mean ± SD 8.39 ± 1.43 years) or baseline refractive error (+0.13 ± 0.27 DS). At initial encounters, most clinicians monitored without intervention (mean across cohort years 91.9%), with some recommending lifestyle changes (3.5%) and SV spectacles/CL (3.0%). This pattern remained stable over the years studied. Pre-myopic children developed myopia at a similar age over the study period (mean ± SE: 9.66 ± 0.16 years) and experienced a faster rate of loss of hyperopic reserve (loss of −0.26 ± 0.07 D/year in the 2017 cohort vs. −0.73 ± 0.18 D/year in the 2020 cohort and −0.71 ± 0.10 D/year in the 2021 cohort) regardless of patient age. **Conclusions**: Pre-myopic children in the 2020 and 2021 cohort years experienced an accelerated loss of hyperopic reserve compared to those in the 2017 cohort. Despite this, very few pre-myopic children were recommended lifestyle changes, which were known to be effective for delaying myopia onset. Since delaying myopia onset may be more impactful than subsequent myopia treatment, additional research should focus on effective interventions for the pre-myopic population.

## 1. Introduction

Myopia is a growing public health concern worldwide, with global prevalence rising rapidly, especially among children and adolescents [1,2]. Estimates suggest that myopia will increase from roughly 30% of the world’s population in 2020 to 40–50% by 2050, with high myopia affecting about 10% [3,4]. A more recent systematic review and meta-analysis predict that by 2050, myopia will affect almost 40% of children and adolescents globally, representing over 740 million young people [4]. The development of myopia at a young age is particularly concerning as these cases often progress more rapidly, resulting in a higher degree of myopia and greater axial elongation by adulthood [5,6,7,8,9,10]. Any level of myopia increases the lifetime risk of irreversible vision loss, with high myopia posing the greatest risk due to complications that may affect central and peripheral vision [11]. Beyond the vision implications, this burden translates into reduced quality of life, decreased educational and occupational opportunities, and increased dependency and contributes to significant long-term economic costs [12,13,14].

Identifying young children at risk of developing myopia and intervening early to delay or prevent its onset are critical [6]. Delaying the onset of myopia has a substantial long-term impact. In East Asian children, each year that the onset of myopia is postponed is associated with 0.68–0.97D less myopia by adulthood, reflecting the equivalent of several years of active myopia control. Similarly, in non-East Asian children, delaying its onset results in a reduction of 0.23–0.50D per year by adulthood [15]. These findings reinforce the importance of early, proactive management of children at risk of myopia development.

Pre-myopia has been defined by the International Myopia Institute (IMI) as a refractive state of ≤+0.75D and >−0.50D in children, combined with other measurable risk factors predicting future myopia [16]. A lower-than-expected hyperopic refractive error, termed the hyperopic reserve, has been associated with a higher likelihood of developing myopia [17]. Children who develop myopia experience accelerated axial growth, starting up to three years before the onset of myopia and continuing for five years after, with the fastest elongation occurring in the year prior to myopia development [18]. The IMI-Clinical Management Guidelines Report emphasizes the importance of identifying pre-myopic children early, enabling clinicians to consider interventions to prevent or delay myopia development [19]. In practice, the guideline suggests a comprehensive analysis of risk factors, including a detailed history of family myopia, ethnicity, and the visual environment, as well as conducting baseline ocular measurements, including cycloplegic refraction, binocular vision assessment, and, where available, axial length, followed by regular monitoring and timely intervention selection.

Appropriate identification of pre-myopic children enables early intervention. Lifestyle interventions such as increasing outdoor time and reducing near-work activities have been supported by research for more than ten years [20,21,22,23,24,25]. Greater outdoor exposure significantly lowers the risk of myopia onset in a dose-dependent manner [26]. A Canadian study demonstrated that each additional weekly hour of time spent outdoors reduced the risk of becoming myopic by ~14% [27]. Pre-myopic children require a minimum of 120 min of outdoor exposure per day to mitigate refractive shifts towards myopia [28]. Likewise, increased near work has been associated with increased myopia incidence. One study revealed that children exposed to higher amounts of near work were more likely to become myopic compared to those who completed less near work [29]. A meta-analysis found that each additional hour per day of screen time (a subset of near work) is associated with 21% higher odds of myopia [30].

A few studies have used existing myopia control interventions, such as low-dose atropine, low-level red light, and myopia control spectacles, to evaluate their efficacy in preventing myopia in pre-myopic children. A recent meta-analysis found that low-level red light therapy and low-dose atropine are most effective in limiting refractive change toward myopia, reducing axial elongation, and reducing myopia incidence in pre-myopic children [31]. However, some concerns remain regarding the use of low-level red light therapy, as these devices may exceed retinal safety limits, underscoring the need for further investigation [32]. Myopia control spectacles are emerging as a low-risk option for pre-myopes, showing promise in slowing progression towards a myopic refractive error as well as reducing axial elongation [33,34,35].

Optometrists play a critical role in recognizing and managing pre-myopic children. In Canada, access to myopia management interventions has expanded significantly over the past decade. A recent study of optometrists in Ontario found a slow but steady increase in the adoption of myopia control management strategies for myopic children between 2017 and 2021, reflecting a growing alignment of clinical practice with emerging evidence and international guidelines [36]. However, it remains unknown how optometrists are managing pre-myopic children in Canadian clinical practices, as well as how these approaches have evolved over time.

Through a retrospective chart review, the purpose of the present study was to determine how optometrists in Ontario, Canada, were managing their young pre-myopic patients. The study examined the extent to which optometrists in Ontario recommended lifestyle changes, such as increasing outdoor time or reducing near work, or prophylactic interventions such as low-dose atropine or myopia control spectacles. Temporal trends in myopia development were evaluated based on patients who progressed from pre-myopia to myopia.

## 2. Methods

The research methodology for this study has been previously described [36]. To summarize, optometry practices across Ontario, Canada, were invited to participate in a general chart-review research study. Of the interested practices, 15 practices were enrolled, with a preference for those that indicated being active in the provision of eye care to children. The retrospective review included charts for children aged 6–10 who presented with a refraction (dry or with cycloplegia) of ≤−0.50D (myopes) or ≤+0.75D to >−0.50D (pre-myopes) and had a comprehensive eye exam between 2017 and 2021. Inclusion and exclusion criteria are detailed in Table 1. A patient’s first visit was defined as the first captured visit within the study period of 2017 to 2021. Patient charts were randomly sampled to ensure balanced representation across ages and visit years. Up to five unique patient charts were selected for each age (age: 6, 7, 8, 9, and 10), visit year (2017, 2018, 2019, 2020, and 2021), and group (myopes and pre-myopes), for a maximum of 250 unique patient charts per practice.

The chart review captured demographic information, refractive error data, and details of the refractive management strategy for each patient. Data from all subsequent visits for each patient were included. Management options consisted of no intervention recommended, single-vision (SV) correction (SV spectacles or SV contact lenses (CL)), bifocal or progressive spectacles, vision therapy, ortho-keratology (ortho-K), atropine, myopia control spectacles, myopia control CL, lifestyle changes (increasing outdoor time and reducing screen time), and myopia control discussion. The study protocol was approved by the institutional ethics committee at the University of Waterloo. Study data were collected and managed using REDCap, an electronic data capture tool hosted at the University of Waterloo. The study was conducted under a Waiver of Informed Consent. The study was designed to be in accordance with the ethical principles in the Declaration of Helsinki and the ICH guidelines for Good Clinical Practice (GCP).

### Statistical Methods

Statistical analyses were performed using JASP software (version 0.17.1, Amsterdam, The Netherlands). Homogeneity of variance was determined by Levene’s test. To standardize follow-up periods across cohort years, the analyses of myopia trends (i.e., age of myopia onset and rate of hyperopic reserve loss) were constrained to the shortest available follow-up period. Pre-myopia management strategies were analyzed across all visits using an analysis of variance (one-way: cohort year; two-way: year x intervention) with post hoc pairwise *t*-tests with Bonferroni correction. Data that were not normally distributed were analyzed with the Kruskal–Wallis test, along with post hoc pairwise Dunn’s tests. Statistical significance was determined as *p* < 0.05.

## 3. Results

### 3.1. Demographics

**Figure 1:**
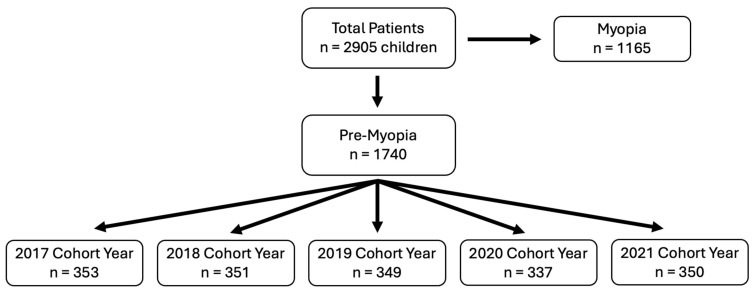
Distribution of pre-myopic patients by cohort year, defined as the year of their first visit within the study period of 2017–2021. The retrospective chart review identified 2905 eligible participants, of which 1740 children were pre-myopic (≤+0.75D to >−0.50D).

A total of 2905 patient charts (*n* = 1467 female) were included in this study, encompassing 8546 visits from 15 practices across Ontario, Canada. Data were extracted consecutively from practices between May 2022 and February 2024. Patient visits spanned January 2017 to February 2024. The results from myopic patients were previously published [36]. The focus of this manuscript is the pre-myopic patients (*n* = 1740), encompassing 4090 visits. Of these patients, 1153 (66.2%) had multiple documented visits. Cycloplegia was seldomly used, amounting to only 1.64% of visits across all visit years (67/4090 visits, for a total of 56 unique patients). Patients were sorted into cohort years based on the year of their first visit between 2017 and 2021 at each practice: 2017 cohort, *n* = 353; 2018 cohort, *n* = 351; 2019 cohort, *n* = 349; 2020 cohort, *n* = 337; and 2021 cohort, *n* = 350 (Figure 1). Cohort year groups did not differ in baseline age (mean ± SD: 8.39 ± 1.43 years, F_4,1735_ = 0.16, *p* > 0.05) or baseline refractive error (mean ± SD: +0.13 ± 0.27 DS, F_4,1735_ = 0.19, *p* > 0.05). Demographic details of pre-myopic patients and those who subsequently became myopic during the time period of the study can be found in Table 2.

### 3.2. Age of Myopia Onset and Loss of Hyperopic Reserve

**Figure 2:**
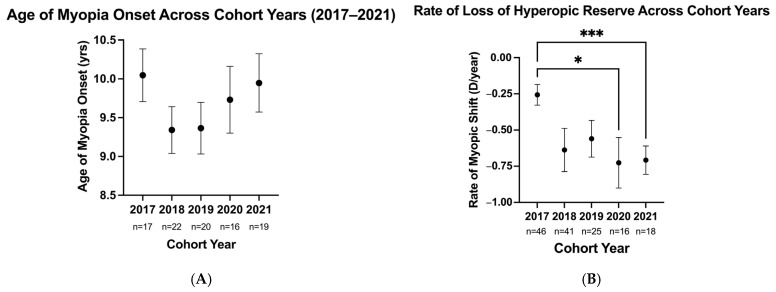
Age of myopia onset (**A**) and yearly rate of change in hyperopic reserve (**B**) for pre-myopic children with incident myopia across the years studied (2017 to 2021) within a standardized 2-year window. While the analysis of myopia onset was limited to patients who developed myopia within this window, the rates of hyperopic reserve loss were computed prior to myopia onset. * denotes *p* < 0.05; *** denotes *p* < 0.0005.

One hundred and eighty-four (184) pre-myopic patients developed myopia during the years studied (10.6%). To ensure comparable follow-up periods, myopia development trends were computed based on the shortest available follow-up period (2-year window for the 2021 cohort year). While the analysis of myopia onset was limited to patients who developed myopia within a 2-year window, the rates of hyperopic reserve loss were computed prior to myopia onset within this standardized window (see sample sizes in Figure 2A,B). Pre-myopic children developed myopia at a similar age over the cohort years (mean ± SE: 9.66 ± 0.16 years, H_4_ = 3.2, *p* > 0.05; Figure 2A). Pre-myopic children in the 2020 and 2021 cohorts experienced a faster rate of loss of hyperopic reserve than the 2017 cohort, regardless of patient age (loss of −0.26 ± 0.07 D/year in the 2017 cohort vs. −0.73 ± 0.18 D/year in the 2020 cohort and −0.71 ± 0.10 D/year in the 2021 cohort, H_4_ = 20.56, *p* = 0.0004; Figure 2B).

### 3.3. Management of Pre-Myopia

#### 3.3.1. Initial Visits

**Figure 3:**
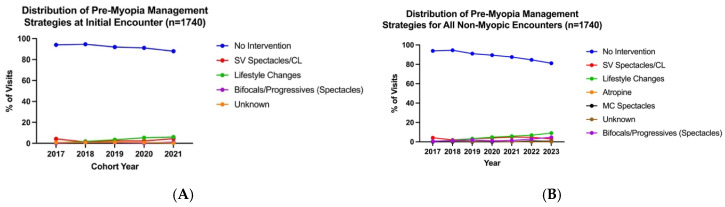
Distribution of pre-myopia management strategies at initial encounter (**A**) and all subsequent non-myopic encounters (**B**).

At initial encounters for all pre-myopic patients (*n* = 1740), the majority of clinicians monitored without intervention (mean across cohort years: 91.9%), with some recommending lifestyle changes (3.5%). SV spectacles/CL (3.0%) or bifocals/progressives (0.8%) were seldom prescribed to address accommodative and binocular vision abnormalities. Management was not documented for 0.8% of initial visits. This distribution of initial management strategies remained stable over the years studied (F_4,16_ = 0.000003, *p* > 0.05; Figure 3A), though recommendations of lifestyle changes increased modestly from 0.57% of visits in 2017 to 6% in 2021.

#### 3.3.2. Subsequent Visits

Pre-myopia management strategies for all subsequent visits were also analyzed, and we excluded visits after myopia onset. From 2022, 0.025% atropine (*n* = 3 patients) and myopia control spectacles (*n* = 7 patients) were recommended by seven different clinicians across 15 visits. Nevertheless, most clinicians continued to monitor without intervention (decreasing from 93.9% of visits in 2017 to 81.1% in 2023), while a few recommended lifestyle changes (increasing from 0.6% of visits in 2017 to 9.0% by 2023). This distribution of subsequent management strategies remained stable over the years studied (F_6,36_ = 0.0012, *p* > 0.05; Figure 3B).

**Figure 4:**
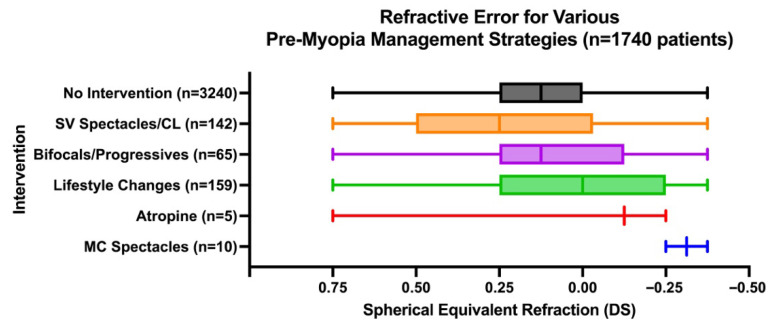
Box plot of patient refractive error across the different modalities used in the management of pre-myopic patients (*n* = 1740 patients, 3643 visits). Box boundaries denote the upper and lower quartiles, with the line within the box representing the median. Whiskers represent the full range of data points. Quartile ranges cannot be displayed for atropine and myopia control (MC) spectacles due to the limited sample size (*n* = 3 and 7 patients respectively). The number of visits that an intervention was recommended is listed on the left (i.e., the same patient may have been recommended the same or different interventions at subsequent visits).

Patient age did not influence the choice of the pre-myopia management strategy (*p* > 0.05). However, the recommended pre-myopia management strategy differed by refractive error (F_5,3615_ = 8.52, *p* < 0.0001, Figure 4). The mean spherical equivalent refraction (SER) was marginally less hyperopic in children who received recommendations for lifestyle changes (+0.05 DS) than in those monitored without intervention (+0.14DS) (t_3615_ = 3.78, *p* = 0.0024). Another statistically significant comparison showed that the mean SER of children who were prescribed lifestyle changes (+0.05DS) was also less hyperopic than those who were prescribed SV spectacles/CL (+0.23 DS, t_3615_ = 5.23, *p* < 0.0001) and bifocals/progressives (+0.19 DS, t_3615_ = 3.34, *p* = 0.013). Atropine (*n* = 3 patients; five visits total) was recommended at a mean SER of −0.08 DS (range: from +0.75 to −0.375), while myopia control spectacles (*n* = 7 patients; 10 visits total) were recommended at a mean SER of −0.16 DS (range: from +0.50 DS to −0.375 DS).

The average recommended follow-up period was 12.1 ± 4.0 months. The most common follow-up periods were 12 months (recommended at 82.3% of visits), 24 months (5.9%), then 6 months (3.9%). Four of the 15 practices had an instrument capable of measuring axial length, though it was primarily used with children who were already myopic. Only five of the pre-myopic 487 children (1%) in these practices had an axial length measurement taken. Of these five children, three had subsequent follow-up visits scheduled every 6 months, a pattern that started in October 2021 and continued through to 2023.

## 4. Discussion

This retrospective chart review revealed that clinicians in Ontario, Canada, most commonly chose a “watch and wait” management approach for their pre-myopia patients. This pattern persists despite evidence of the efficacy of interventions to delay or prevent the onset of myopia. A recent systematic review concluded that “Low-level red light therapy and low-dose atropine are the most effective, generally safe strategies for preventing myopia in at-risk children without myopia, while a non-invasive approach, outdoor activities, provides moderate benefits” [31]. Of note, low-level red light therapy is not an available option for Canadian optometrists. Initiating interventions in pre-myopic children can be more impactful than treating established myopia. Delaying the onset of myopia by even 1 year may yield a long-term benefit equivalent to ~2–3 years of treatment with currently available myopia control modalities [15]. Furthermore, there are published clinical guidelines that include recommendations for pre-myopic intervention [19].

While it is understandable that the use of active interventions such as atropine or myopia control spectacles may not yet be widely supported due to a lack of longitudinal efficacy data as well as possible side effects, recommending lifestyle changes, including increasing outdoor time and limiting near tasks, remains reasonable given the minimal cost and potential for meaningful benefit. Practitioners may still be waiting for evidence from longitudinal studies in order to routinely recommend myopia control for pre-myopic children.

The treatment of pre-myopia falls under the category of primary prevention, which is defined as “an intervention that aims to reduce the risk of disease before it begins by targeting modifiable risk factors in individuals who are still healthy” [37]. In part, this reluctance to initiate treatment is understandable, as pre-myopic children typically have no visual complaints and demonstrate good visual acuity. However, practitioners and parents must recognize the risk of progression to myopia along with its associated long-term ocular complications.

The low adoption of preventative strategies by practitioners and patients is pervasive in healthcare. Patients may hesitate for a variety of psychological and behavioral reasons, including a “wait and see” mentality, the invisibility of prevention outcomes, procrastination, immediate costs, and mistrust of either the clinician’s motivations or the healthcare system as a whole [38]. Practitioners have their own set of reasons for not promoting prevention. These include reimbursement models that favor treating conditions rather than prevention, training that focuses on acute care, time constraints, knowledge of the condition, and a lack of training/skills in prevention presentation [39].

Insights on preventing myopia at the pre-myopic stage can be derived from the approach used in the prevention of diabetes, especially in patients diagnosed as having pre-diabetes. In both situations, early identification and the promotion of meaningful lifestyle changes are critical for reducing future disease risk. In a systematic review of diabetes prevention [40], Messina et al. reported that a patient’s willingness to modify lifestyle habits, together with their understanding of diabetes and its long-term consequences, strongly influenced prevention outcomes. Motivation provided by a trusted healthcare professional also positively impacted outcomes. However, barriers such as difficulties in modifying established lifestyle routines, a lack of time, financial constraints, and personal challenges hindered the adoption of prevention strategies. Professional factors such as clinician workload, knowledge of the disease, willingness and comfort in providing advice, and confidence in the ability to deliver diabetes prevention advice also play a large role in the implementation of preventative services. Enhancing practitioner competence and improving patient understanding are critical for effective implementation of preventative measures. Similar barriers faced by both patients and practitioners are likely influencing the management of pre-myopia. Enhancing practitioner confidence through clearer clinical guidelines and improved training, along with strengthening caregivers’ understanding of pre-myopia and its potential long-term implications, will likely be critical for increasing the adoption of early preventative strategies in pediatric eye care.

Patients may also respond differently to recommendations depending on whether the proposed strategy involves lifestyle modification versus a more active intervention strategy. For example, Danish adults at risk of cardiovascular disease demonstrated an overwhelming preference for lifestyle changes over medication [41]. This insight may be valuable when counseling caregivers of pre-myopic children. Identifying time outdoors as a lifestyle choice versus an active intervention choice (the fitting of myopia prevention glasses or contact lenses) may guide the decision-making process.

Important trends in the progression toward myopia were observed in our sample across the years studied. Pre-myopic children examined in the years 2020 and 2021 experienced an accelerated loss of hyperopic reserve compared with pre-myopic children seen in 2017. Similar trends were recently reported in a cohort of children in China, with a reported shift toward earlier onset of myopia [42]. This trend is concerning, as earlier myopia onset is consistently associated with greater axial elongation and an increased risk of high myopia in adulthood [8,9,10,43].

In this cohort, the average recommended follow-up interval for pre-myopic children was relatively long, averaging 12.1 ± 4.0 months, with the majority of clinicians (82.3%) recommending annual reviews. However, it is known that the most rapid rate of change occurs between the year preceding myopia onset and the onset year itself, indicating that this period represents a distinct phase of accelerated eye growth, ultimately highlighting the importance of close monitoring during this critical period [18]. Current expert guidance recommends monitoring pre-myopic children at least every six months to detect early refractive and/or axial changes associated with an increased risk of progression [44,45]. Only 3.9% of visits documented a 6-month follow-up recommendation, reflecting limited uptake of proactive monitoring.

Although four of the 15 participating practices had access to instrumentation to measure axial length, an objective measure recognized as highly informative in assessing progression risk in pre-myopia cases [46], only five of the 487 pre-myopic children seen in these four practices had a documented axial length measurement. The limited use of axial length assessments, combined with the predominant recommendation of annual follow-up visits, highlights a missed opportunity for earlier detection of progression toward myopia and timely implementation of prevention strategies.

Nonetheless, retrospective chart-review study designs have several limitations. Since only 66.2% of our sample had multiple documented visits, loss to follow-up may affect estimates of myopia development. Chart reviews may not capture the full extent of discussions with patients and parents, as documentation varies by clinician and recording style (paper vs. electronic records). It is possible that a discussion of myopia prevention occurred but was not recorded. Known risk factors for myopia development, such as information on parental myopia, were not explicitly recorded on 74% of pre-myopic patient charts. As a result, it was not possible to determine whether clinicians managed pre-myopic children who may be at higher risk differentially. While it is possible that clinicians were also aware of a child’s parental myopia history, for example, if parents are existing patients but did not explicitly document this in the patient chart, it is unclear whether this relevant information is being considered when determining management strategies. Similarly, we found that cycloplegia was rarely used in determining the refractive error, and axial length was seldomly measured in pre-myopic children. Incorporating cycloplegia to control accommodation during refractive assessments and monitoring axial length as a quantitative indicator would support a more comprehensive assessment of myopia risk in clinical practice. Non-cycloplegic measurements may have resulted in an overestimation of myopia and hyperopic reserve loss. Finally, these results may not be generalizable to other provinces and countries, as children in Ontario have comparatively greater access to eye care, as pediatric eye examinations are covered under the provincial healthcare plan, which may influence detection rates, follow-up patterns, and management decisions.

## 5. Conclusions

This study highlights a critical misalignment between the changing trends of myopia development and the limited adoption of preventative strategies and objective monitoring in pre-myopic children. As the burden of myopia continues to grow worldwide, it is essential to encourage proactive preventative management in continuing education programs for clinicians and to develop clearer evidence-based guidelines for the management of pre-myopic patients to mitigate future myopia development.

## Figures and Tables

**Table 1:** Inclusion and exclusion criteria.

Category	Inclusion and Exclusion Parameters
Age	6–10 years old
Appointment time frame	≥1 appointment between 2017 and 2021
Spherical Equivalent Refraction (SER)	Pre-Myopia: −0.25 to +0.75 DS Myopia: ≤−0.50 DS
Cylinder	≤−1.00 DC
Binocular vision	No strabismus or amblyopia
Best-corrected visual acuity (BCVA)	≥20/30 in each eye
Other	No ocular or systemic disease; no developmental abnormalities

DS = dioptre sphere; DC = dioptre cylinder.

**Table 2:** Demographic characteristics of pre-myopic patients.

	Pre-Myopic Group	Pre-Myopic Patients Who Became Myopic
*n*	1740	184
Gender	854/1740 = 49.1% female	98/184 = 53.3% female
Mean age at first visit (SD); Range	8.39 (1.43) years;	8.39 (1.45) years;
	6.0–10.9 years	6.0–10.9 years
Spherical equivalent refraction (SER) at first visit (SD); Range	+0.13 (0.27) DS;	−0.04 (0.27) DS;
	+0.75 to −0.375 DS	+0.75 to −0.375 DS
Parental Myopia	Neither	Neither
	307/1740 = 17.6%	35/184 = 19.0%
	One Parent	One Parent
	97/1740 = 5.6%	18/184 = 9.8%
	Both Parents	Both Parents
	47/1740 = 2.7%	14/184 = 7.6%
	Unknown	Unknown
	1289/1740 = 74.1%	117/184 = 63.6%

SD = standard deviation.

## Data Availability

The dataset (comprising clinical records) presented in this article is not readily available to preserve patient privacy. Access to the dataset may be granted upon reasonable request to the corresponding author, subject to institutional review and data sharing agreements.
